# A Case Study: Incidental Finding of Human Intestinal Spirochetosis in Screening Colonoscopy

**DOI:** 10.7759/cureus.55422

**Published:** 2024-03-03

**Authors:** Bhovineey Ramanathan, Vinod Ramachandran, Abdul Rana, Christopher McDonald

**Affiliations:** 1 Surgery, Lyell Mcewin Hospital, Adelaide, AUS; 2 Colorectal Surgery, Northern Adelaide Local Health Network, Adelaide, AUS

**Keywords:** his, human intestinal spirochetosis (his), colonoscope, fobt, spirochetosis, human intestinal spirochetosis

## Abstract

Human intestinal spirochetosis (HIS) is a rare occurrence. We present an interesting case study on an asymptomatic over-60-year-old male who was incidentally discovered to have HIS following a colonoscopy that was conducted for his positive fecal occult blood test (FOBT). Histopathology of the colonic biopsy proved the presence of human intestinal spirochetosis; however, as he was asymptomatic, treatment was not initiated in his case. We discuss here the prevalence, presentation, diagnostic methods, and treatment of colonic HIS.

## Introduction

Human intestinal spirochetosis (HIS) is frequently related to Brachyspira aalborgi and Brachyspira pilosicoli infections [1]. There is very little literature available describing the pathogenic relevance of these microorganisms to humans. Brachyspira are anaerobic bacteria that grow slowly despite being fastidious. There has also been reported difficulty in isolating them and growing them in laboratory conditions [2]. HIS is an uncommon clinicopathological state distinguished by the existence of spiral-shaped microorganisms on the surface of the colorectal mucosa, and occasionally, by their infiltration into the lamina propria [3]. How these spirochetosis are transmitted to humans remains inadequately understood, and there is an ongoing debate regarding whether they function as pathogens or as commensal residents. However, research has indicated that fecal-oral transmission is a possible mode of transmission [4].

## Case presentation

We present an interesting case study of an over-60-year-old male who was referred following a positive fecal occult blood test (FOBT). He has a background of dyslipidemia and gout but no other significant medical history. He was asymptomatic otherwise and reported no overt per rectal (PR) bleed, no diarrhea, no weight loss, and no other constitutional symptoms. He reported no family history of malignancy, and this was his index colonoscopy. Physical examination was unremarkable, and no abnormalities were detected. He was booked for an urgent colonoscopy in view of the positive fecal occult blood test (FOBT).

Moderate diverticulosis mainly in the sigmoid colon was noted in the colonoscopy. There were patchy areas of inflammation noted from the right colon to the distal transverse colon. Random and targeted biopsies of the whole colon were taken. Second-degree hemorrhoids were also noted, which were banded at the end of the procedure. The patient tolerated the procedure well and was subsequently discharged with an outpatient follow-up in the clinic arranged to chase the histopathology report.

**Figure 1:**
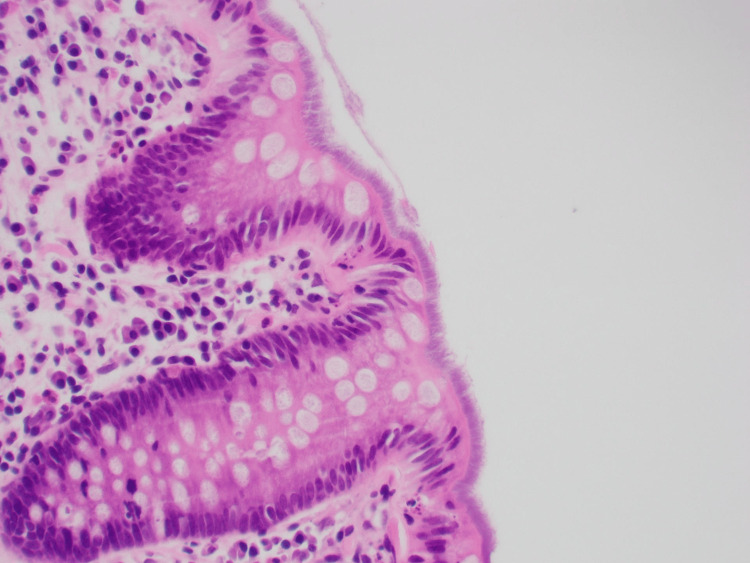
Histology of the colonic biopsy revealing surface basophilic filaments in keeping with intestinal spirochetosis

**Figure 2:**
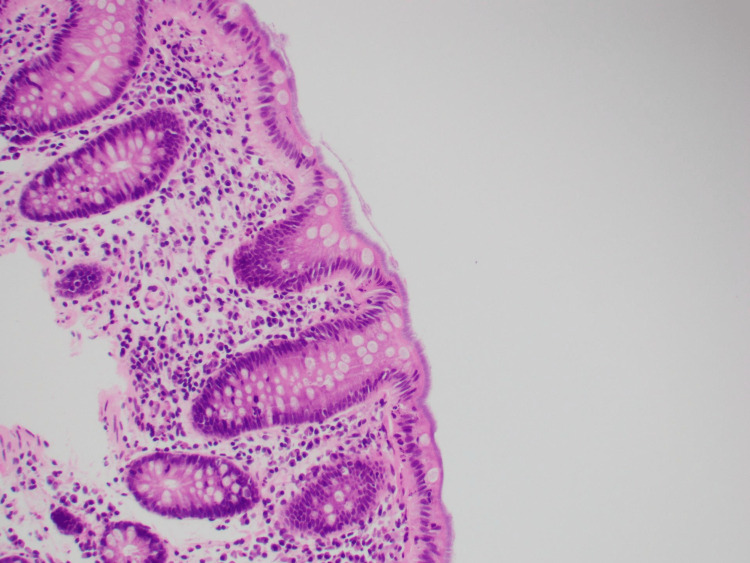
Histology of the colonic biopsy in keeping with intestinal spirochetosis

The histopathology of the colonic biopsy (Figure 1) from the colonoscope revealed surface basophilic filamentous in keeping with intestinal spirochetosis (IS). The left colon had some mild focal active colitis with the manifestation of the disease but no significant inflammation was identified in the right colon. The lamina propria was mildly edematous and contained the usual mononuclear cell population with occasional lymphoid aggregates. A layer of blue filamentous organisms was seen on the mucosal surface, in keeping with intestinal spirochetosis. Attached are the images of the biopsy. Figure 1 shows the histology of the colonic biopsy, revealing surface basophilic filamentous in keeping with intestinal spirochetosis, and Figure 2 shows another part of the biopsy concordant with intestinal spirochetosis.

The range of potential differential diagnoses is quite extensive and encompasses several conditions, including (i) irritable bowel syndrome (IBS), (ii) inflammatory bowel diseases like Crohn's disease, (iii) microscopic colitis, and (iv) malabsorptive syndromes like celiac disease, chronic pancreatitis, and lactose intolerance. The surprising factor is that our patient did not exhibit any symptoms throughout. Chronic gastrointestinal infections are often associated with diarrhea, with common microbial culprits, including Clostridium difficile, Vibrio cholerae, Salmonella, Shigella, and Giardia, but our patient was asymptomatic throughout. However, in rare instances, the presence of spirochetes colonizing the colon can lead to chronic diarrhea and other associated symptoms.

## Discussion

Human intestinal spirochetosis was first discovered in the 1960s by Harland and Lee [1]. The distinctive presence of colonic spirochetosis on the epithelial surface, which is discerned through a standard histological analysis of biopsies obtained during colonoscopy is the gold standard of the diagnosis [2]. The incidence of human intestinal spirochetosis is low but varies between 2.5% and 32.5%) and is usually present in the rectum of patients infected with human immunodeficiency virus (HIV) [3]. The prevalence of HIS is higher in West Africa, Southern India, and Asia [4]. Risk factors include immunodeficient states and homosexuality [5]. Frequently, HIS is identified in the screening colonoscopy of patients presenting with vague abdominal pain and chronic watery diarrhea. Colonoscopy findings are generally not specific; they appear either as a normal mucosa, polypoid lesion, or erythematous area [6].

The conventional method of diagnosis involves the histological assessment of colonic biopsy samples [7]. Microorganisms are typically visualized using the hematoxylin-eosin (H&E) stain and observed through light microscopy. In this process, the abnormality becomes apparent on the epithelial surface, characterized by thread-like structures arranged in a palisade-like fashion, often referred to as the "blue fringe."

Human colonic spirochetosis tends to colonize from the proximal to the distal colon, including the vermiform appendix [6]. Intestinal spirochetosis has been uncommonly found in the stomach and small intestine since the 1900s [8,9]. A study in Japan showed HIS has a preference for the right colon [10]. Alsaigh and Fogt conducted a study involving 15 biopsies that confirmed the presence of intestinal spirochetosis through histological examination [6]. Their findings revealed diverse endoscopic manifestations of colonic spirochetosis [11], which included a polyp-like appearance in seven patients, a singular lesion in one patient, and an erythematous region in another patient [9]. A case report presented by Kostman and colleagues detailed a scenario in which a patient with acquired immunodeficiency syndrome (AIDS) exhibited widespread colonic ulceration [12]. Furthermore, for additional clarification, alternative staining techniques, such as Warthin-Starry and Dieterle silver impregnation, can be employed. When examined under electron microscopy, spirochetosis tends to embed itself within colonic cells without invading them. These spirochetosis are observed attaching to the cell membrane, typically within areas where microvilli have been damaged or destroyed [13].

The severity of the patient’s symptoms and their clinical presentation should guide the decision on whether to treat intestinal spirochetosis or not. IS may manifest either without symptoms, as in our patient, given that the organisms believed to be responsible maintain a commensal relationship with the normal gut flora, or with symptoms related to gastrointestinal issues, as these organisms can also take on an invasive and pathogenic nature [10]. For patients without symptoms, a conservative approach can be considered using the ‘wait-and-watch’ principle. Symptomatic patients benefit from monotherapy, metronidazole (500 mg 4 times a day for 10 days) [9]. It outperforms the use of macrolides, clindamycin, and a combination of macrolides with metronidazole. In situations involving co-infection, a combination of antibiotic therapies might be necessary. The dosage and duration of treatment can vary, contingent on factors like age and body weight [14].

Unfortunately, there have been some reported relapse of symptoms in patients treated with metronidazole on follow-up. This is likely attributed to re-infection or incomplete eradication [15]. Research conducted by Jabbar and colleagues revealed that metronidazole treatment had the paradoxical effect of encouraging the relocation of Brachyspira into the colonic crypts and goblet cell granules [15]. This observation suggests a potential bacterial strategy for evading antibiotic exposure [16].

## Conclusions

In a nutshell, intestinal spirochetosis is an uncommon cause of abdominal pain and diarrhea in immunocompetent patients. Although it usually happens in HIV patients, there are more reports available on its existence in healthy individuals. Biopsy via an endoscopic procedure is the main method of diagnosis. Treatment of these patients depends on the patient’s clinical presentation and the severity of its manifestation. For our patient, since he did not exhibit any symptoms, it was best to employ the ‘wait-and-watch principle’ and treat him if he happens to develop any symptoms in the future.

## References

[1] Tsinganou E, Gebbers JO (2010). Human intestinal spirochetosis--a review. Ger Med Sci.

[2] Fan K, Eslick GD, Nair PM (2022). Human intestinal spirochetosis, irritable bowel syndrome, and colonic polyps: a systematic review and meta-analysis. J Gastroenterol Hepatol.

[3] Petruzzellis C, Catino F (2023). Unusual behavior of intestinal spirochetosis: a case report. Medicine (Baltimore).

[4] Yang M, Lapham R (1997). Appendiceal spirochetosis. South Med J.

[5] Alnimer L, Zakaria A, Warren B (2021). A case of human intestinal spirochetosis diagnosed during screening colonoscopy. Cureus.

[6] Alsaigh N, Fogt F (2002). Intestinal spirochetosis: clinicopathological features with review of the literature. Colorectal Dis.

[7] van Mook WN, Koek GH, van der Ven AJ, Ceelen TL, Bos RP (2004). Human intestinal spirochaetosis: any clinical significance?. Eur J Gastroenterol Hepatol.

[8] Tong YT, Younes M (2020). Intestinal spirochetosis: case series and review of the literature. Ann Clin Lab Sci.

[9] Graham RP, Naini BV, Shah SS, Arnold CA, Kannangai R, Torbenson MS, Lam-Himlin DM (2018). Treponema pallidum Immunohistochemistry is positive in human intestinal Spirochetosis. Diagn Pathol.

[10] Pérez-Tanoira R, Tamarit MD, Montaña AM (2023). Increased prevalence of symptomatic human intestinal spirochetosis in MSM with high-risk sexual behavior in a cohort of 165 individuals. Trop Med Infect Dis.

[11] Körner M, Gebbers JO (2003). Clinical significance of human intestinal spirochetosis--a morphologic approach. Infection.

[12] Kostman JR, Patel M, Catalano E, Camacho J, Hoffpauir J, DiNubile MJ (1995). Invasive colitis and hepatitis due to previously uncharacterized spirochetes in patients with advanced human immunodeficiency virus infection. Clin Infect Dis.

[13] Lee FD, Kraszewski A, Gordon J, Howie JG, McSeveney D, Harland WA (1971). Intestinal spirochaetosis. Gut.

[14] Novick SD, Berhanu M, Negassi YG, Demissie SW, Hussain Kazmi SA, Holder SS (2023). Intestinal spirochetosis and chronic diarrhea: a case report and literature review. Cureus.

[15] Jabbar KS, Dolan B, Eklund L (2021). Association between Brachyspira and irritable bowel syndrome with diarrhoea. Gut.

[16] Mikosza AS, La T, Brooke CJ (1999). PCR amplification from fixed tissue indicates frequent involvement of Brachyspira aalborgi in human intestinal spirochetosis. J Clin Microbiol.

